# Dietary Patterns in Children with Attention Deficit/Hyperactivity Disorder (ADHD)

**DOI:** 10.3390/nu6041539

**Published:** 2014-04-14

**Authors:** Hae Dong Woo, Dong Woo Kim, Young-Seoub Hong, Yu-Mi Kim, Ju-Hee Seo, Byeong Moo Choe, Jae Hong Park, Je-Wook Kang, Jae-Ho Yoo, Hee Won Chueh, Jung Hyun Lee, Min Jung Kwak, Jeongseon Kim

**Affiliations:** 1 Molecular Epidemiology Branch, National Cancer Center, 323 Ilsan-ro, Ilsandong-gu, Goyang-si, Gyeonggi-do 410-769, Korea; E-Mails: eastsea93@hanmail.net (H.D.W.); ellebass@gmail.com (D.W.K.); 2 Department of Preventive Medicine, College of Medicine, Dong-A University, Dong-A University Hospital, 26, Daesingongwon-ro, Seo-gu, Busan 602-715, Korea; E-Mails: yshong@dau.ac.kr (Y.-S.H.); kimyumi@dau.ac.kr (Y.-M.K.); 3 Heavy Metal Exposure Environmental Health Center, Dong-A University, 32, Daesingongwon-ro, Seo-gu, Busan 602-714, Korea; E-Mail: juhui978@dau.ac.kr; 4 Department of Psychiatry, College of Medicine, Dong-A University, Dong-A University Hospital, 26 Daesingongwon-ro, Seo-gu, Busan 602-715, Korea; E-Mails: bmchoe@dau.ac.kr (B.M.C.); pjhkorea@hanmail.net (J.H.P.); 5 Department of Child and Adolescent Psychiatry, College of Medicine, Inje University Busan Paik Hospital, 75 Bokji-ro, Busanjin-gu, Busan 614-735, Korea; E-Mail: forevery99@hanmail.net; 6 Department of Pediatrics, College of Medicine, Dong-A University, Dong-A University Hospital, 26 Daesingongwon-ro, Seo-gu, Busan 602-715, Korea; E-Mails: pedendo@dau.ac.kr (J.-H.Y.); hwchueh@dau.ac.kr (H.W.C.); 7 Department of Pediatrics, Kosin University Gospel Hospital, 262, Gamcheon-ro, Seo-gu, Busan 602-702, Korea; E-Mail: agasoa@hanmail.net; 8 Department of Pediatrics, Pusan National University Hospital, Pusan National University School of Medicine, 179, Gudeok-ro, Seo-gu, Busan 602-739, Korea; E-Mail: glory0123@hanmail.net

**Keywords:** dietary pattern, attention deficit/hyperactivity disorder (ADHD), school-aged children, Korean

## Abstract

The role of diet in the behavior of children has been controversial, but the association of several nutritional factors with childhood behavioral disorders has been continually suggested. We conducted a case-control study to identify dietary patterns associated with attention deficit hyperactivity disorder (ADHD). The study included 192 elementary school students aged seven to 12 years. Three non-consecutive 24-h recall (HR) interviews were employed to assess dietary intake, and 32 predefined food groups were considered in a principal components analysis (PCA). PCA identified four major dietary patterns: the “traditional” pattern, the “seaweed-egg” pattern, the “traditional-healthy” pattern, and the “snack” pattern. The traditional-healthy pattern is characterized by a diet low in fat and high in carbohydrates as well as high intakes of fatty acids and minerals. The multivariate-adjusted odds ratio (OR) of ADHD for the highest tertile of the traditional-healthy pattern in comparison with the lowest tertile was 0.31 (95% CI: 0.12–0.79). The score of the snack pattern was positively associated with the risk of ADHD, but a significant association was observed only in the second tertile. A significant association between ADHD and the dietary pattern score was not found for the other two dietary patterns. In conclusion, the traditional-healthy dietary pattern was associated with lower odds having ADHD.

## 1. Introduction

Attention deficit hyperactivity disorder (ADHD) is one of the most commonly diagnosed neurobehavioral disorders in childhood, and it often lasts into adulthood [1]. ADHD prevalence rates vary by age, gender, and ethnicity [2,3]. Boys are more likely to have ADHD than girls, and higher rates of ADHD in younger age groups have been observed in studies of children and adolescents [4]. Worldwide, the overall prevalence of ADHD/hyperkinetic disorder (HD) was found to be 5.29% in a pooled analysis [2]. The prevalence of ADHD is 8.7% in US children aged eight to 15 years [5] and 9.7% in Iranian school-aged children [6]. In Korea, the prevalence of ADHD is 7.6% in elementary school children with a mean age of 9.4 years [7] and upper-grade elementary school children with a mean age of 11.6 years [8]. The etiology of ADHD is multifactorial, and both genetic and environmental factors may be involved in ADHD [9]. Family and twin studies have shown that genes play an important role in the development of ADHD. Genome-wide association studies are inconclusive, but candidate gene studies suggest the involvement of genes related to the receptors and transporters of dopamine and serotonin [10,11]. Proposed ADHD environmental risk factors include heavy metal and chemical exposures such as lead, mercury, organochlorine, organophosphates, and phthalates, as well as nutritional and lifestyle/psychosocial factors [5].

The effect of diet and dietary supplements is unclear, but considerable evidence suggests that dietary factors are associated with childhood behavioral disorders such as ADHD [12,13]. Low levels of copper, iron, zinc, magnesium, and omega-3 fatty acids have been reported in children with ADHD, and sugar, artificial food colorings, and preservatives are associated with an increased risk of ADHD [12,13]. Recently, the association between dietary pattern and ADHD has been examined in several studies [6,20,21]. As nutrients are consumed in combination and because nutrients are highly interrelated, the study of dietary patterns is useful to further understand the overall role of diet in ADHD. Thus, the purpose of this study was to determine the association between various dietary patterns and ADHD among Korean school-aged children.

## 2. Experimental Section

### 2.1. Study Population

We conducted a hospital-based case-control study using elementary school students who visited several university hospitals in Busan, Korea, from April to September, 2013. ADHD cases were recruited from two university hospitals (Dong-A and Inje University). ADHD was diagnosed by psychiatrists based on the Diagnostic and Statistical Manual of Mental Disorders-Fourth Edition (DSM-IV). Some children with ADHD have concurrent condition such as tic disorder (motor type), anxiety disorder, oppositional defiant disorder, Tourette’s disorder, depression, and learning disability. A total of 117 cases, which consented to participate in research, were recruited, and age- and sex-matched controls were recruited from three university hospitals (Dong-A, Pusan, and Kosin University). Controls who did not have severe chronic diseases, a history of ADHD diagnosis and any related disease, such as mental disorder and tic disorder were recruited. Additional test using ADHD Rating Scale (ARS) for controls was performed to exclude ADHD cases. After excluding seven participants who did not complete the questionnaire, a total of 202 controls were recruited. To exclude the seasonal variation in dietary intake, the dietary survey season was also matched in the analysis. Frequency matching by grade (two years), sex, and season (three months) was conducted. A total of 192 elementary school students aged seven to 12 years (96 students with ADHD and 96 healthy controls) were finally selected. Each participant and their legal guardian were provided with an informed consent form according to the procedures approved by the Institutional Review Board of the National Cancer Center.

### 2.2. Data Collection

The legal guardians of the participants were asked to complete a self-administered questionnaire, which was used to gather information on demographics, lifestyle, and the medical histories of the participants and their parents. A trained interviewer facilitated the 24-h recalls (24HR) interviews face-to-face, and another two non-consecutive 24HR interviews were conducted by telephone between April and September 2013. Individual food intake was calculated using CAN-PRO 4.0 (Computer Aided Nutritional Analysis Program, The Korean Nutrition Society, Seoul, Korea). Mercury and lead exposure from food was calculated using dietary consumption data and their concentrations in 118 core food items. Consumption of omega-3 fatty acids was estimated as the sum of eicosapentaenoic acid (EPA) and docosahexaenoic acid (DHA).

### 2.3. Statistical Analysis

Principal-components analysis (PROC FACTOR) was used to extract the participants’ dietary patterns using 32 predefined food groups. We used a varimax rotation to enhance the interpretability of the analyzed factors. We determined how many factors to retain after evaluating the eigenvalue, scree test, and interpretability. The dietary patterns were named according to the factors with the highest scores among the defined food groups for each dietary factor. Each dietary pattern’s factor score was categorized by tertile for further analysis. Using a Student *t*-test for continuous variables and a chi-square test for categorical variables, we compared the general characteristics between students with ADHD and controls. The trend test was performed to analyze the associations between each of the dietary patterns and ADHD using a generalized linear model with adjustments for total energy intake. Odds ratios (ORs) and 95% confidence intervals (CIs) for ADHD were calculated across the tertiles of dietary pattern scores using logistic regression models. The lowest tertile of each dietary pattern was used as the reference. To assess the trend across the tertiles, we assigned median values to each tertile of the dietary pattern scores as a continuous variable. We performed the statistical analysis using SAS version 9.2 (SAS Institute Inc., Cary, NC, USA). All *P* values were two-tailed (α = 0.05).

## 3. Results

The general characteristics of the study population are presented in Table 1. The mean ages of the controls and students with ADHD were 9.1 and 9.0 years, respectively. The total energy intake was higher in controls than in students with ADHD (*p* = 0.008). Father’s educational background and occupation significantly differed between ADHD students and controls (*p* < 0.001 and *p* = 0.001, respectively).

**Table 1:** General characteristics of study population ^1^.

Characteristics	Controls (*n* = 96)	Cases (*n* = 96)	*P*
Age (year)	9.1 ± 1.8	9.0 ± 1.7	
Sex, male (%)	65 (67.7)	65 (67.7)	
Total energy intake (kcal)	2027.3 ± 381.7	1879.2 ± 380.7	0.008
Weight (kg)	34.3 ± 9.6	33.1 ± 10.4	0.427
Body mass index (cm/m^2^)	18.2 ± 2.9	17.5 ± 3.2	0.122
Gestation age (week)	39.0 ± 1.7	39.0 ± 1.7	0.787
Birth weight (kg)	3.3 ± 0.5	3.2 ± 0.5	0.099
Breastfeeding, yes (%)	80 (83.3)	70 (72.9)	0.081
Mother’s age (year)	38.6 ± 3.7	39.5 ± 4.1	0.133
Birth order	1.5 ± 0.7	1.4 ± 1.1	0.651
Father’s education, *n* (%)			
<High school	22 (22.9)	45 (47.9)	<0.001
College	53 (55.2)	41 (43.6)	
Graduate school	21 (21.9)	8 (8.5)	
Father’s occupation, *n* (%)			
Professional	26 (27.1)	15 (15.6)	0.001
Office/service worker	44 (45.8)	28 (29.2)	
Manual worker	13 (13.5)	28 (29.2)	
Other	13 (13.5)	25 (26.0)	
Father’s smoking status, *n* (%)			
Seldom or Never	37 (39.0)	24 (26.4)	0.188
Current	42 (44.2)	49 (53.9)	
Former	16 (16.8)	18 (19.8)	

^1^ All analyses were performed with the data matched for age, sex, and dietary survey season.

PCA identified four major dietary patterns among the 32 food groups, and the associated factor loading scores with absolute values ≥0.20 are shown in Table 2. The “traditional” dietary pattern was characterized by high intakes of condiments, vegetables, tofu/soymilk, and mushrooms. The “seaweed-egg” dietary pattern included high intakes of seaweeds, fats/oils, sweets, and eggs. The “traditional-healthy” dietary pattern included high intakes of kimchi, grains, bonefish, and low intakes of fast foods and beverages. The “snack” dietary pattern was characterized by high intakes of snacks and processed meat and a low intake of noodles. Lean fish, other seafood, and yogurt were not listed due to their low factor loadings in all examined dietary patterns. Each dietary pattern explained 8.0%, 6.0%, 5.6%, and 5.4% of the variation in food intake, respectively.

**Table 2:** Factor loadings for the four major dietary patterns derived from principal components analysis with orthogonal rotation.

Foods/Food Groups	Traditional	Seaweed-Egg	Traditional-Healthy	Snack
Condiments	0.75			
Vegetables	0.56		0.20	
Tofu, Soymilk	0.53			
Mushrooms	0.49			
Salted fermented seafood	0.34			
Fruits	0.32	−0.22		−0.31
Seaweeds		0.69		
Fats, Oils	0.29	0.68		
Sweets	0.27	0.43		0.33
Egg		0.41		
Potatoes	0.22	0.35		
Processed fruit products		0.33		
Legumes		0.29		
Kimchi			0.58	−0.23
Grains	0.23		0.56	
Bonefish	0.28		0.52	0.26
Fatty fish	0.29		0.23	−0.38
Snack				0.49
Processed meats				0.44
Bread				0.43
Milk				0.30
Shellfish			−0.22	
Beverages		0.22	−0.44	
Fast foods			−0.49	
Rice cake		−0.36		
Seeds	0.23			
Dairy products			−0.23	−0.23
Meats			−0.31	−0.31
Noodles		0.24		−0.49
Variance of explained (%)	8.0	6.0	5.6	5.4

Factor loadings with absolute values ≥0.20 were listed in the table among 32 food groups.

The distribution of characteristics by dietary pattern score tertiles is presented in Table 3. Increasing scores in the traditional and traditional-healthy patterns were correlated with a decreased percent energy from fat (*P* for trend = 0.001; *P* for trend <0.001, respectively), whereas the percent energy from carbohydrate increased as the score of the traditional-healthy pattern increased (*P* for trend <0.001). Fatty acids were significantly associated with dietary pattern scores. The traditional pattern score was associated with a high intake of total fatty acids; the seaweed-egg and traditional-healthy pattern scores were associated with high intakes of PUFAs and omega-3 fatty acids, whereas the snack pattern score was negatively associated with the intakes of total fatty acids, PUFAs, and MUFAs. Regarding mineral intake, calcium intake was positively associated with the scores of the traditional, traditional-healthy, and snack patterns, and iron was positively associated with the scores of the traditional and traditional-healthy patterns. Heavy metal exposure via food consumption was also assessed, and mercury was positively associated with the traditional, traditional-healthy, and snack patterns; lead was positively associated with the traditional and snack patterns.

The ORs and 95% CIs of ADHD were analyzed across the tertiles of dietary pattern scores (Table 4). The OR (95% CI) in the highest tertiles of the traditional dietary pattern compared to those in the lowest tertiles in crude model was 0.29 (0.13–0.64), but a significant association was not observed in multivariate model 2 (OR: 0.76, 95% CI: 0.26–2.24). The seaweed-egg pattern was not significantly associated with ADHD in any of the models. The snack pattern score was positively associated with the risk of ADHD, but a significant association was observed only in the second tertile in crude model and multivariate model 1. Students in the highest tertile of the traditional-healthy pattern score had an increased risk of ADHD in the multivariate-adjusted models when compared with those in the lowest tertile (OR (95% CI): 0.32 (0.13–0.82) in multivariate model 1; 0.31 (0.12–0.79) in multivariate model 2).

**Table 3:** Distribution of characteristics by the tertiles of dietary pattern scores.

**Characteristics**	**Traditional**				**Seaweed-Egg**			
	**T1 ^1^**	**T2**	**T3**	***P* Trend ^2^**	**T1**	**T2**	**T3**	***P* Trend**
Age (year)	8.7 (1.8)	9.1 (1.7)	9.5 (1.6)	0.014	8.9 (1.6)	9.0 (1.8)	9.2 (1.8)	0.270
Sex, female (%)	32 (43.2)	19 (25.7)	11 (25.0)	0.023	28 (40.6)	22 (33.9)	12 (20.7)	0.018
BMI (kg/m^2^)	17.9 (3.3)	17.6 (3.0)	18.3 (2.7)	0.533	17.6 (2.5)	17.7 (3.1)	18.4 (3.6)	0.171
Education, ≥college (%)	41 (56.9)	51 (68.9)	31 (70.5)	0.109	41 (59.4)	40 (63.5)	42 (72.4)	0.132
Total energy intake (kcal)	1757.7 (353)	2005.6 (325)	2194.2 (383)	<0.001	1898.9 (418)	1897.2 (362)	2080.8 (352)	0.008
Carbohydrate (g)	248.6 (44.7)	284.5 (49.5)	315.4 (59.2)	0.013	273.0 (61.6)	272.3 (51.4)	289.5 (53.9)	0.216
Carbohydrate (% energy)	56.6 (6.5)	56.5 (6.2)	57.0 (4.9)	0.057	57.4 (6.3)	57.1 (6.0)	55.3 (5.5)	0.173
Protein (g)	67.1 (22.3)	78.2 (14.4)	86.0 (15.2)	0.198	71.0 (18.7)	74.0 (21.6)	83.2 (15.3)	0.016
Protein (% energy)	15.0 (3.0)	15.6 (2.2)	15.6 (1.7)	0.075	14.9 (2.2)	15.4 (2.8)	16.0 (2.2)	0.005
Fat (g)	56.9 (19.1)	63.3 (18.4)	68.1 (18.4)	<0.001	59.7 (20.6)	59.4 (18.2)	67.3 (17.4)	0.951
Fat (% energy)	28.3 (5.1)	27.9 (5.1)	27.4 (4.4)	0.001	27.7 (5.3)	27.6 (4.9)	28.7 (4.6)	0.792
Total fatty acids (g)	29.2 (12.8)	33.1 (14.2)	33.2 (12.6)	0.031	28.9 (13.9)	30.9 (12.7)	35.7 (12.7)	0.080
PUFAs (g)	6.9 (2.5)	8.7 (3.4)	8.5 (2.7)	0.798	6.4 (2.6)	7.8 (2.1)	10.1 (3.2)	<0.001
MUFAs (g)	10.9 (5.7)	12.2 (5.9)	12.4 (5.3)	0.057	10.8 (5.9)	11.5 (5.4)	13.1 (5.5)	0.245
Omega-3fatty acids (g)	0.10 (0.26)	0.23 (0.48)	0.34 (0.62)	0.134	0.25 (0.57)	0.20 (0.38)	0.15 (0.38)	0.059
Calcium (mg)	491.5 (222)	587.7 (171)	730.0 (224)	0.002	565.1 (225)	558.1 (230)	632.8 (206)	0.755
Iron (mg)	10.4 (2.3)	13.5 (4.8)	16.6 (8.4)	<0.001	12.2 (7.3)	12.8 (5.3)	14.1 (3.4)	0.447
Zinc (mg)	8.2 (2.1)	9.8 (2.0)	10.5 (1.9)	0.112	8.9 (2.2)	9.1 (2.3)	10.2 (1.9)	0.063
Mercury (µg/kg bw)	0.19 (0.05)	0.22 (0.06)	0.22 (0.07)	0.027	0.20 (0.06)	0.21 (0.06)	0.21 (0.06)	0.965
Lead (µg/kg bw)	0.43 (0.14)	0.50 (0.14)	0.53 (0.17)	0.022	0.48 (0.17)	0.47 (0.13)	0.49 (0.16)	0.954
**Characteristics**	**Traditional-Healthy**				**Snack**			
	**T1**	**T2**	**T3**	***P* Trend**	**T1**	**T2**	**T3**	***P* Trend**
Age (year)	9.2 (1.7)	8.8 (1.8)	9.1 (1.7)	0.746	9.8 (1.7)	8.8 (1.7)	8.8 (1.7)	0.002
Sex, female (%)	31 (41.9)	18 (29.0)	13 (23.2)	0.022	13 (25.0)	34 (42.5)	15 (25.0)	0.906
BMI (kg/m^2^)	17.8 (3.1)	17.8 (3.0)	18.0 (3.3)	0.644	19.5 (3.7)	17.4 (2.3)	17.0 (2.8)	<0.001
Education, ≥college (%)	47 (63.5)	41 (67.2)	35 (63.6)	0.956	32 (61.5)	52 (66.7)	39 (65.0)	0.719
Total energy intake (kcal)	1946.2 (411)	1893.1 (391)	2029.3 (343)	0.225	2088.5 (361)	1812.7 (362)	2023.5 (387)	0.354
Carbohydrate (g)	266.5 (56.5)	272.8 (52.1)	298.1 (55.8)	<0.001	292.7 (58.3)	260.5 (53.4)	287.7 (52.8)	0.566
Carbohydrate (% energy)	54.6 (6.0)	57.7 (5.9)	58.3 (5.5)	<0.001	55.6 (6.1)	57.2 (6.3)	56.9 (5.5)	0.326
Protein (g)	77.4 (23.4)	71.8 (17.3)	77.9 (15.2)	0.294	82.6 (15.6)	71.2 (21.4)	75.8 (18.1)	0.079
Protein (% energy)	15.7 (3.1)	15.1 (1.9)	15.2 (1.9)	0.293	15.7 (2.4)	15.5 (2.8)	14.8 (1.9)	0.047
Fat (g)	65.7 (20.5)	58.5 (19.2)	60.6 (16.3)	<0.001	67.7 (18.0)	56.0 (17.9)	64.7 (19.8)	0.872
Fat (% energy)	29.7 (4.6)	27.2 (5.0)	26.5 (4.6)	<0.001	28.7 (5.1)	27.3 (5.0)	28.2 (4.6)	0.843
Total fatty acids (g)	30.4 (12.9)	30.6 (12.1)	34.3 (15.1)	0.244	38.3 (16.9)	28.3 (10.0)	30.3 (11.9)	0.001
PUFAs (g)	7.6 (3.1)	7.7 (2.8)	8.8 (3.0)	0.046	9.1 (3.2)	7.5 (2.5)	7.7 (3.3)	0.018
MUFAs (g)	11.3 (5.5)	11.4 (5.2)	12.8 (6.4)	0.300	14.8 (7.5)	10.4 (4.0)	10.9 (4.7)	<0.001
Omega-3fatty acids (g)	0.13 (0.34)	0.18 (0.36)	0.33 (0.63)	0.024	0.29 (0.66)	0.19 (0.38)	0.14 (0.31)	0.124
Calcium (mg)	553.3 (227)	551.3 (195)	658.0 (231)	0.016	555.1 (221)	541.2 (206)	663.5 (227)	<0.001
Iron (mg)	12.0 (4.1)	12.8 (5.0)	14.6 (7.6)	0.026	13.8 (3.8)	12.0 (4.6)	13.7 (7.8)	0.669
Zinc (mg)	9.3 (2.5)	9.2 (2.1)	9.7 (2.0)	0.909	10.1 (2.1)	8.7 (2.1)	9.6 (2.3)	0.293
Mercury (µg/kg bw)	0.19 (0.05)	0.21 (0.06)	0.23 (0.06)	0.003	0.19 (0.06)	0.21 (0.06)	0.22 (0.06)	0.001
Lead (µg/kg bw)	0.45 (0.13)	0.49 (0.15)	0.50 (0.18)	0.125	0.44 (0.16)	0.47 (0.14)	0.51 (0.16)	0.006

^1^ Tertiles of dietary pattern scores; ^2^ *P* trend was calculated using generalized linear models for continuous variables and using Mantel–Haenszel chi-squared tests for categorical variables; *P* trend of nutrient and metal consumption was adjusted for total energy intake; PUFAs: Polyunsaturated fatty acids, MUFAs: Monounsaturated fatty acids.

**Table 4:** Distribution of characteristics by the tertiles of dietary pattern scores ^1^.

Dietary Pattern		*N* Control/Case	Crude Model	Multivariate Model 1 ^2^	Multivariate Model 2 ^3^
Traditional	T1 ^4^	32/42	1	1	1
	T2	32/42	1.00 (0.52–1.92)	1.32 (0.61–2.84)	1.88 (0.80–4.42)
	T3	32/12	0.29 (0.13–0.64)	0.43 (0.18–1.04)	0.76 (0.26–2.24)
	*P* trend ^5^		0.003	0.072	0.615
Seaweed-egg	T1	32/37	1	1	1
	T2	32/33	0.89 (0.45–1.76)	0.66 (0.30–1.44)	0.70 (0.31–1.55)
	T3	32/26	0.70 (0.35–1.42)	0.64 (0.29–1.41)	0.84 (0.36–1.94)
	*P* trend		0.321	0.271	0.682
Traditional-healthy	T1	32/42	1	1	1
	T2	32/30	0.71 (0.36–1.41)	0.60 (0.27–1.32)	0.57 (0.25–1.29)
	T3	32/24	0.57 (0.28–1.15)	0.32 (0.13–0.77)	0.31 (0.12–0.79)
	*P* trend		0.113	0.011	0.014
Snack	T1	32/20	1	1	1
	T2	32/48	2.40 (1.17–4.91)	2.93 (1.22–7.05)	2.34 (0.95–5.79)
	T3	32/28	1.40 (0.66–2.98)	1.69 (0.70–4.07)	1.59 (0.65–3.91)
	*P* trend		0.571	0.451	0.505

^1^ All analyses were performed with the data matched for age, sex, and dietary survey season; ^2^ Adjusted for gestation age, birth weight, mother’s age, birth order, father’s education, and father’s occupation; ^3^ Model 2 + additional adjustment for total energy intake, omega-3 fatty acids, lead, and mercury consumption; ^4^ Tertiles of dietary pattern scores; ^5^ Tests for trend were conducted by assigning the median value to each tertile of heavy metal intake as a continuous variable.

## 4. Discussion

The present study identified four dietary patterns. The traditional-healthy dietary pattern, characterized by high intakes of kimchi, grains, and bonefish, and low intakes of fast foods and beverages, was associated with lower odds having ADHD. Although the present study focused on dietary factors, significant associations with ADHD were found in father’s education and occupation. Socioeconomic status of children is generally related to household income, and parent’s educational background and occupation. Children from lower socioeconomic status are more likely diagnosed with ADHD than children from higher socioeconomic status in previous studies [14,15,16,17]. Family income [14,15], parent’s education [15,16,17] and occupation [15,16] were significantly associated with ADHD. Education status of mother was highly correlated with that of fathers in this study, and occupation of mother did not vary compared to that of father’s. Thus, fathers’ educational background and occupation were used as surrogate of socioeconomic status. As those variables were high associated with ADHD, we adjusted them for the analysis.

The role of diet in the behavior of children has been controversial, but associations between several nutritional factors and child behavior such as ADHD have been continually suggested [12,13]. Food additives, sugar, and aspartame are considered negative factors in the development of ADHD, and thus, dietary intervention studies with special diets, including additive-free and sugar elimination diets, have been conducted. A meta-analysis has reported that artificial food coloring is associated with childhood hyperactivity [18]. However, in a sugar elimination intervention study, there was no evidence that refined sugar affected child behavior [19,20,21,22,23,24].

The role of polyunsaturated fatty acids (PUFAs), particularly omega-3 fatty acids, in relation to neurodevelopmental disorders has been studied because omega-3 fatty acids play a critical role in brain development and function [25]. Children with ADHD have lower levels of omega-3 fatty acids, and the supplementation of omega-3 fatty acids can reduce the symptoms of ADHD in school-aged children and adolescents [26,27]. However, there was no clear evidence of improvement in ADHD symptoms with omega-3 supplementation in randomized controlled trials, but these findings could be the result of methodological problems [28,29]. The association between dietary pattern score and fatty acid intake was investigated in this study. The traditional, seaweed-egg, and traditional-healthy pattern scores were negatively associated with ADHD, although only the traditional-healthy pattern had a statistically significant association; moreover, they were positively associated with fatty acid intake. By contrast, the snack pattern score showed a positive association with ADHD and was negatively associated with the intake of total fatty acids, PUFAs, and MUFAs. However, additional adjustment for omega-3 fatty acid intake did not change the statistically significant association between the traditional-healthy dietary pattern and ADHD. Thus, the factors associated with the beneficial effects of a healthy dietary pattern might be complex.

Regarding mineral intake, calcium was positively associated with the scores of the traditional, traditional-healthy, and snack patterns, and iron was positively associated with the scores of the traditional and traditional-healthy patterns. Zinc was not associated with any of the four pattern scores. Iron deficiency may be associated with ADHD [30] because iron stores in the brain can influence dopamine-dependent functions [31,32]. A case-control study in India reported that the serum ferritin level was lower in children with ADHD [33], while another study found that ADHD symptoms in children with low serum ferritin levels were alleviated following iron supplementation [34]. In a 19-year follow-up study, the iron status of Costa Rican children was found to be associated with behavioral problems in adolescents [35]. The role of zinc nutrition in ADHD is not clear, but evidence suggests that zinc is beneficial in the treatment of children with ADHD [36,37]. Zinc deficiency is involved in dopamine transporter dysfunction [38], and intervention studies have found that zinc supplementation can reduce ADHD symptoms in children with low zinc levels [39,40,41]. Both, low iron and zinc levels have been associated with dopamine metabolism, and low levels of iron and zinc are involved in impaired dopamine transmission in subjects with ADHD [42,43,44,45].

Heavy metal exposure via food consumption was also investigated. Mercury was positively associated with the traditional, traditional-healthy, and snack patterns, and lead was positively associated with the traditional and snack patterns. The association between lead exposure and ADHD has been widely studied, and a meta-analysis has reported that lead exposure is positively associated with ADHD symptoms [46]. In a study with school-aged children living in two Romanian cities near a metal-processing plant, an association with ADHD was observed only for lead exposure, not aluminum or mercury exposure [47]. An association between the blood mercury level and ADHD in Chinese children in Hong Kong has been observed [48], but a significant association was not found in a cross-sectional study of Romanian children [47] or in a Children’s Health and Environment Research (CHEER) study that surveyed elementary schools in six South Korean cities [49,50]. A more clear association with ADHD has been observed for lead exposure, even at low concentrations [49,50]. Prenatal mercury exposure is associated with an increased risk of neurobehavioral disorders, and lead exposure in childhood has been associated with ADHD [51]. In this study, lead and mercury consumption was positively correlated with the traditional-healthy dietary pattern, but it did not alter the beneficial effects of the traditional-healthy dietary pattern on ADHD.

Recently, associations between dietary patterns and ADHD have been examined in several cross-sectional studies [6,20,21]. One study, which included a population-based cohort of adolescents, reported that a Western-style dietary pattern, characterized by high intakes of fat, refined sugars, and sodium and low intakes of fiber, folate, and omega-3 fatty acids, was associated with increased odds of an ADHD diagnosis, whereas a healthy dietary pattern, with high intakes of fiber, folate, and omega-3 fatty acids, was not correlated with the diagnosis of ADHD [20]. In a study of adolescents in China, three major dietary patterns were identified, and dietary patterns characterized by a high intake of snacks or animal-derived foods were associated with higher odds for psychological symptoms [21]. In Iranian school-aged children, four major dietary patterns were identified. The higher scores of the dietary patterns associated with a high intake of sweets and fast food were associated with greater odds for having ADHD, but no significant association was observed for the healthy or Western dietary patterns [6]. In this study, traditional-healthy dietary pattern was positively associated with dietary factors, such as PUFAs and minerals that are known for beneficial effects on ADHD. Another beneficial effect of the traditional-healthy dietary pattern might be associated with the low fast food intake. Junk foods are generally high in fat, sugar, additives, artificial food colorings, and preservatives, which may negatively affect ADHD symptoms [52]. Overall, the traditional-healthy dietary pattern was associated with many dietary factors that affect childhood behavioral disorders, such as ADHD.

The present study has several limitations. As this was a case-control study, it is possible that dietary intake was affected by an individual’s health status and social background. Thus, causal inference cannot be determined. Results could differ by ADHD types, but information about ADHD type was not gathered for subgroup analysis due to small sample size. However, such pattern analyses are useful to further understand the diet of ADHD children as a whole rather than classifying it by a single nutrient or food group.

## 5. Conclusions

The traditional-healthy dietary pattern, which is characterized by high intakes of kimchi, grains, and bonefish, and low consumption of fast foods and beverages, appears to be negatively associated with ADHD in school-aged Korean children.
